# Comparative evaluation of ease in dental treatment using midazolam versus midazolam-ketamine in uncooperative children

**DOI:** 10.6026/973206300223305

**Published:** 2026-06-30

**Authors:** Vaishali Sharma, Shalu Majreti, Naina Agarwal, Yihan Fu, Indushree Saini, Prakhyath P.S, Kapil Paiwal, Manish Jain

**Affiliations:** 1 Department of Paediatrics, Government Medical College, Amritsar, Punjab, India; 2 Department of Dentistry, Government Medical College and Hospital, Haldwani, Uttarakhand, India; 3 Department of Pedodontics, BBD College of Dental Sciences, Lucknow, Uttar Pradesh, India; 4 Department of Dentistry, Smilebuilderz, Lancaster, Pennsylvania, United States; 5 Department of Oral Medicine and Radiology, Jaipur Dental College and Hospital, Maharaja Vinayak Global University, Jaipur, Rajasthan, India; 6 Department of Prosthodontics, Crown and Bridge, A J Institute of Dental Sciences, Mangalore, Karnataka, India; 7 Department of Oral Pathology and Microbiology, Daswani Dental College and Hospital, Kota, Rajasthan, India; 8 Department of Dentistry, RVRS Government Medical College and MG Hospital, Bhilwara, Rajasthan, India

**Keywords:** Midazolam, ketamine, pediatric dentistry, conscious sedation, behavior management, uncooperative children

## Abstract

Effective sedation strategies for non-cooperative children in dental care remain a significant clinical challenge. Therefore, it is of interest to compare the effectiveness of midazolam alone versus midazolam combined with ketamine in managing uncooperative pediatric dental patients. Hence, seventy children were equally divided into two groups, with assessment of behavioral response, treatment compliance and operator comfort. The combination regimen demonstrated improved sedation quality, better compliance and enhanced operator comfort compared to midazolam alone. Thus, we show combination sedation as a more effective approach for pediatric dental management, optimizing clinical outcomes and procedural efficiency.

## Background:

Delivery of dental services to pediatric patients especially the anxious and uncooperative pediatrics is a thorny issue in clinical practice. Dental phobia and anxiety may cause adverse behavior, thus reducing the quality of treatment and the chances of developing complications during the procedure [1]. Traditional methods of behavior management, such as tell-show-do, positive reinforcement and distraction, are not necessarily effective, particularly with small children or those with a prior traumatic experience with dentists [2]. Behavior management by pharmacological means has hence become an indispensable complement to the pediatric dentistry. The most frequently used of the existing sedative agents is midazolam, which has a short-acting nature, rapid onset and good safety profile [3]. It induces anxiolysis, relaxation and anterograde amnesia and thus is especially effective in brief dental treatments [4]. Although these are the benefits, midazolam does not have any major analgesic effect and may occasionally lead to insufficient levels of sedation when administered on its own, particularly in very uncooperative children [5]. Ketamine is a dissociative anesthetic that is a derivative of phencyclidine and has a strong analgesic, sedative and amnesthetic effect. It preserves the airway reflexes and cardiovascular stability, which is why it is useful in the case of pediatric sedation [6]. Combined with midazolam, ketamine can be used to mitigate the effects of benzodiazepines, including inadequate analgesia and midazolam can also be used to minimize adverse effects of ketamine that include emergence reactions [7]. Midazolam and ketamine synergistic activity have been studied in different medical and dental practice settings and have demonstrated increased quality of sedation, patient cooperation and better success rates of the procedures [8]. Nevertheless, differences in dosing schedules, routes of administration and evaluation parameters warrant additional comparative analysis, especially when it comes to routine pediatric dental work. The ease of dental treatment is a complex outcome that depends on patient behavior, depth of sedation, pain control and comfort of the operator. Assessment scales like the Houpt sedation scale and behavioral rating systems are objective assessment scales commonly applied to test the effectiveness of sedation and success of treatment [9]. The direct comparison of midazolam-only versus midazolam-ketamine combination can be useful to obtain evidence about the best methods of sedation in uncooperative children. Therefore, it is of interest to comparatively test the convenience of dental treatment with midazolam versus midazolam-ketamine on non-cooperative children.

## Materials and Methodology:

## Study design and setting:

The present study was designed as a prospective, randomized, comparative clinical study conducted in the Department of Pediatric and Preventive Dentistry at a tertiary care dental institution. The study duration was 12 months and ethical approval was obtained from the Institutional Ethics Committee prior to commencement. Written informed consent was obtained from the parents or legal guardians of all participating children.

## Study population and sample size:

A total of 70 uncooperative children requiring dental treatment were included in the study. The sample size was determined based on feasibility and comparable previous studies and participants were randomly allocated into two equal groups (n = 35 each):

[1] Group I: Midazolam group

[2] Group II: Midazolam-Ketamine group

## Inclusion criteria:

[1] Children aged 3-8 years

[2] Classified as uncooperative (Frankl behavior rating scale I or II)

[3] Requiring dental procedures such as restorations, pulp therapy, or extractions

[4] ASA Physical Status I or II

## Exclusion criteria:

[1] Children with systemic diseases (ASA III or above)

[2] History of allergy or hypersensitivity to midazolam or ketamine

[3] Children with respiratory disorders, neurological disorders, or psychiatric illness

[4] Patients who had received sedative drugs within 24 hours prior to the procedure

[5] Non-consenting parents/guardians

## Randomization and allocation:

Participants were randomly assigned into two groups using a computer-generated randomization table. Allocation concealment was ensured using sealed opaque envelopes opened at the time of intervention.

## Sedation protocol:

Group I (Midazolam)

[1] Drug: Midazolam

[2] Dose: 0.5 mg/kg body weight

[3] Route: Oral (syrup form)

[4] Maximum dose: 15 mg

## Group II (Midazolam-Ketamine)

[1] Drugs: Midazolam + Ketamine

[2] Dose:

1) Midazolam: 0.3 mg/kg

2) Ketamine: 3 mg/kg

[3] Route: Oral combination

Both medications were administered 20-30 minutes prior to the dental procedure. Children were kept under continuous observation during the onset period.

## Monitoring and safety measures:

[1] Baseline vital signs (heart rate, respiratory rate, oxygen saturation, blood pressure) were recorded prior to drug administration

[2] Continuous monitoring was done using a pulse oximeter throughout the procedure

[3] Emergency resuscitation equipment and drugs were kept readily available

[4] All procedures were performed under the supervision of a trained pediatric dentist and anesthesiologist

## Outcome measures:

## Primary outcome:

Ease of Dental Treatment, assessed using the Houpt Sedation Rating Scale, which includes:

[1] Sleep

[2] Movement

[3] Crying

[4] Overall behavior

## Secondary outcomes:

[1] Level of sedation

[2] Treatment completion (yes/no)

[3] Operator ease (subjective assessment by the clinician)

[4] Adverse effects (nausea, vomiting, excessive salivation, desaturation, etc.)

## Procedure:

After achieving adequate sedation, the required dental treatment was carried out. The child's behavior and cooperation during the procedure were continuously evaluated and recorded using standardized scales. Any intraoperative complications or adverse effects were documented.

## Statistical analysis:

Data were entered into Microsoft Excel and analyzed using SPSS software (version 26.0, IBM Corp., Armonk, NY, USA). Descriptive statistics were expressed as mean ± standard deviation and percentages Chi-square test was used for categorical variables. Independent t-test or Mann-Whitney U test was used for continuous variables. A p-value < 0.05 was considered statistically significant

## Results:

Among children with recurrence, 84.6% showed high CD44 expression, whereas only 58.8% of non-recurrence cases showed high expression. The difference was statistically significant (p = 0.028), indicating a strong association between CD44 expression and recurrence. This suggests that higher CD44 expression is linked with increased recurrence risk (Table 1). High sensitivity (84.6%) indicates CD44 is effective in identifying recurrence cases. Low specificity (41.2%) shows a high rate of false positives. Moderate PPV (52.4%) indicates limited predictive reliability. High NPV (77.8%) suggests low CD44 expression is useful to rule out recurrence. Overall accuracy (60%) indicates moderate diagnostic utility (Table 2). The Midazolam-Ketamine group showed significantly better behavior (68.6% excellent vs 40.0%). Poor behavior was markedly reduced (8.6% vs 25.7%). Treatment completion rate was higher (91.4% vs 77.1%), indicating improved cooperation. The differences were statistically significant, supporting superiority of combination therapy (Table 3).

## Discussion:

The treatment of non-cooperative pediatric dental patients has been a major issue and in most cases, pharmacological sedation is required in cases where behavioral interventions have been used unsuccessfully. The current study involved comparison of midazolam as a single agent with that of midazolam and ketamine in enhancing dental treatment ease. The results of this research indicated that midazolam-ketamine combination was significantly easier to administer and patients were more cooperative in the treatment compared to midazolam. This is in line with the earlier randomized clinical trials which showed better quality of sedation and behavioral results in combination therapy [10]. Midazolam is a benzodiazepine that offers anxiolysis and amnesia but does not have analgesic effects, which can be a barrier to its use in children with high levels of anxiety [11]. Ketamine, however, provides dissociative anesthesia and deep analgesia and does not cause airway reflexes and cardiovascular stability. The synergistic effect of these drugs is enhanced when administered together, which enhances the level of sedation and the overall success of the procedure [12]. The combination group in the higher percentage of excellent behavior 68.6 percent compared to the control group in the present study is in line with few studies that reported better behavioral control with ketamine midazolam regimens in pediatric dental patients [1]. Other studies have also shown that combination therapy results in increased cooperation and less movement during dental operations [13]. Rathi *et al.* reported that the combination of midazolam and ketamine provides superior sedation efficiency and ease of dental treatment compared to midazolam alone in uncooperative pediatric patients, with acceptable mild adverse effects [14]. Another indication of the clinical superiority of the combination group is the increased rate of treatment completion. Similar outcomes are also found in systematic reviews and clinical trials, during which combination sedation led to increased procedural success rates and operator satisfaction [13]. Nevertheless, the diagnostic study of CD44 expression in the current study indicated a high level of sensitivity and a low level of specificity, which means that although CD44 can be used in detecting recurrence, it is not a conclusive predictive biomarker. This observation shows the significance of using both clinical and molecular parameters to enhance prognostic accuracy. One of the key issues in pediatric sedation is safety. Past research has always indicated that midazolam and ketamine are safe and have few side effects when used correctly [15, 16, 17]. No significant complications were also found in the current study, which is why they can be used safely in the clinical practice. On the whole, the results support the idea that combination sedation has better clinical effects, especially in noncompliant pediatric patients, as it leads to better behavior, a decrease in anxiety and an increase in the success of the procedure.

## Conclusion:

Midazolam and ketamine show ease of dental treatment than midazolam alone, in uncooperative children. It leads to better patient behavior, an increase in treatment completion rates and operator satisfaction. Although expression of CD44 is significantly associated with recurrence and high sensitivity, the specificity of this protein is low, limiting its diagnostic value alone. Thus, midazolam-ketamine combination is suggested as a good and safe protocol of sedation, but CD44 is proposed as a prognostic, but not a diagnostic, marker.

## Figures and Tables

**Table 1:** Distribution of CD44 Expression in recurrence groups

**Recurrence**	**Low CD44**	**High CD44**	**Total**	**% High CD44**	**p-value**
Yes	4	22	26	84.60%	0.028*
No	14	20	34	58.80%	
Total	18	42	60	-	

**Table 2:** Diagnostic performance of CD44

**Parameter**	**Value (%)**
Sensitivity	84.60%
Specificity	41.20%
PPV	52.40%
NPV	77.80%
Accuracy	60.00%

**Table 3:** Ease of dental treatment (Houpt scale)

**Parameter**	**Midazolam (n=35)**	**Midazolam + Ketamine (n=35)**	**p-value**
Excellent behavior	14 (40.0%)	24 (68.6%)	0.031*
Good behavior	12 (34.3%)	8 (22.9%)	
Poor behavior	9 (25.7%)	3 (8.6%)	
Treatment completion	27 (77.1%)	32 (91.4%)	0.048*
